# Early Diagnosis and Classification of Cerebral Palsy: An Historical Perspective and Barriers to an Early Diagnosis

**DOI:** 10.3390/jcm8101599

**Published:** 2019-10-03

**Authors:** Anna te Velde, Catherine Morgan, Iona Novak, Esther Tantsis, Nadia Badawi

**Affiliations:** 1 Cerebral Palsy Alliance, Discipline of Child and Adolescent Health, Faculty of Medicine and Health, The University of Sydney, New South Wales 2050, Australia; cmorgan@cerebralpalsy.org.au (C.M.); inovak@cerebralpalsy.org.au (I.N.); nadia.badawi@health.nsw.gov.au (N.B.); 2 Discipline of Child and Adolescent Health, Faculty of Medicine and Health, The University of Sydney, New South Wales 2145, Australia; 3 TJ Nelson Department of Neurology & Neurosurgery, The Children’s Hospital at Westmead, New South Wales 2145, Australia; esther.tantsis@health.nsw.gov.au; 4 The Grace Centre for Newborn Intensive Care, The Children’s Hospital at Westmead, Hawkesbury Road, Westmead NSW 2145, Australia

**Keywords:** cerebral palsy, early diagnosis, early detection, classification, motor type, topography, severity, infants, pattern recognition

## Abstract

Since the 1800s, there have been calls in the literature for the early diagnosis of cerebral palsy (CP). However, diagnosis still often occurs late, from 12 to 24 months in high income countries and as late as 5 years in low resource settings. This is after the optimal timeframe for applying interventions which could harness neuroplastic potential in the developing brain. Multiple barriers exist which affect clinicians’ confidence in diagnosing CP early. These range from the lack of definitive biomarkers to a lack of curative treatments for CP. Further barriers to diagnosis are proposed including; (a) difficulty finding a congruent fit with the definition of CP in an infant, where expected activity limitations might not yet be apparent; and (b) differences in the presentation of motor type and topography classifications between infants and children. These barriers may affect a clinicians’ confidence using “pattern recognition” in the differential diagnosis process. One of the central tenets of this paper is that diagnosis and classification are different, involving different instruments, and are more accurately conducted separately in infants, whereas they are fundamentally interconnected in older children and inform therapeutic decisions. Furthermore, we need to be careful not to delay early diagnosis because of the low reliability of early classification, but instead uncouple these two processes. Ongoing implementation of best practice for early detection requires creative solutions which might include universal screening for CP. Implementation and accompanying knowledge translation studies are underway to decrease the average age of diagnosis in CP.

## 1. Background

Definitions of cerebral palsy (CP) have been the subject of debate throughout history. William Little, credited for first describing the condition that is CP in 1861 [1,2], described birth asphyxia as the cause of neurological disturbance in an infant. The term ‘cerebral palsies’ was first coined by William Osler [3], who recognized a heterogeneous group of disorders. In the 2007 consensus definition, the term ‘cerebral palsy’ was defined as “a group of permanent disorders of the development of movement and posture, causing activity limitation, that are attributed to non-progressive disturbances that occurred in the developing fetal or infant brain” [4]. The debate around terminology continues today with the proposition of “cerebral palsy spectrum disorder” now suggested as a more accurate and beneficial term [2]. Barriers exist which undermine a clinician’s confidence in diagnosing CP early. Recognized barriers include: (a) a lack of definitive biomarkers; (b) uneasiness about false positives; (c) the difficult conversation of giving the diagnosis and the resultant grief and perceived stigma; (d) the desire to rule out the differential diagnosis of every treatable condition first; (e) the lack of curative treatments and evidence for early interventions; and (f) making a diagnosis when faced with a lack of definitive signs on traditional clinical examination.

## 2. The Historical Roundabout Calling for the Earlier Diagnosis of CP 

**Figure 1:**
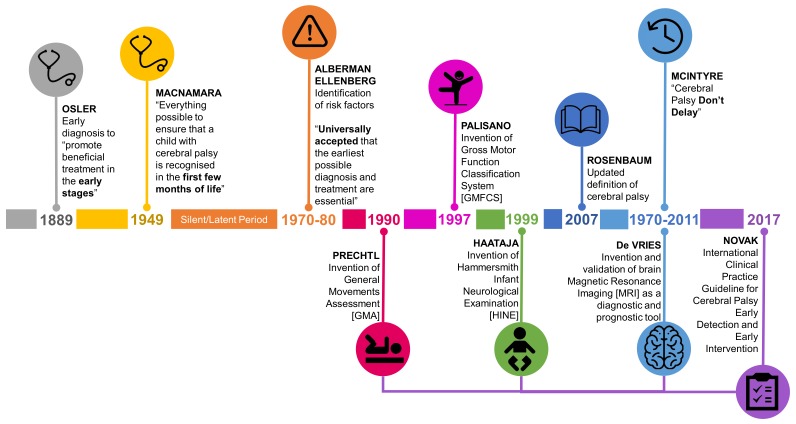
Timeline of calls for early diagnosis of cerebral palsy (CP) including development of evidence based tools with best predictive validity for CP.

Although debate about the definition of CP has followed a trajectory, the discussion about the earliest age at which CP can be accurately diagnosed is a recurrent theme. The notion that early diagnosis is important to ensure early access to disease specific intervention to minimize the impact of CP has been raised in the literature for centuries (Figure 1). As far back as the first description of cases of CP in the 1800s William Little made the argument for earliest possible diagnosis in order to “promote beneficial treatment of the disorders when detected in the early stages” [5] (p. 17). This started the historically recurring argument that earlier identification can lead to earliest possible intervention. However, no actual decrease in the age of diagnosis has accompanied these calls. In the mid-20th century Dr. Jean Macnamara, an Australian female voice, was very clear in questioning if the diagnosis of CP was happening as early as possible: “are we doing everything possible to ensure that a child with cerebral palsy is recognized in the first few months of life? We should insist that in the medical curriculum the medical student is given facilities to recognize cerebral palsy in the baby, and health visitors should be taught how to recognize the signs before the development of deformity” [6] (p. 122). 

Up until the 1970s the concept of early diagnosis of CP in infants was not systematically explored [7]. At this point in history the idea of the “unseen handicap” [8] or “latent or silent” period [9] prevailed. At this time, it was considered that the signs of CP could not be identified in the first 12 months of life. Indeed, it was generally understood, and stressed to clinicians that CP could and should not be diagnosed accurately until the age of 3–5 years of age. During the 1970s and 80s the idea of risk [8] and risk factors [7] for CP were introduced to identify which infants should be followed through the neurologically “silent” period. Again, the call for early diagnosis for access to early intervention persisted. “It is now universally accepted that the earliest possible diagnosis and treatment are essential in order to prevent, or at least minimize, the handicapping effects of a disability and to make the most of the assets a child possesses” [8] (p. 132); “it may be important to identify children who are at substantial risk of CP as early as possible, because therapeutic intervention may offer the best chance of achieving maximal benefit” [7] (p.705).

In the past 50 years there has been an increase in knowledge and tools which accurately identify infants with CP; particularly infants with newborn risks such as prematurity (Figure 1). In the 1970s abnormally retained primitive reflexes were considered a useful clinical sign to identify CP [10,11]; additionally cranial ultrasound (CUS) started to be used in neonatal intensive care units to identify brain lesions [12,13]. The understanding of how neuroimaging modalities, including magnetic resonance imaging (MRI) could be used to predict CP developed from the 1980s [14]. MRI was recommended as a diagnostic tool for CP by the American Academy of Neurology in 2004 [15]. Arguably the biggest leap in the accuracy of predicting CP, and altering the view that infants with CP are neurologically silent in the first few months of life, was the advent of the general movements assessment (GMA); an assessment of spontaneous movement of an infant [16,17,18]. The GMA was first described in the 1990s after many decades of advancing understanding of early spontaneous movements [19]. The Hammersmith Infant Neurological Examination (HINE), a standardized neurological examination, was also developed in the 1990s [20]. These tools started to be pulled together into one body of knowledge in the 2011 seminal article Cerebral Palsy—Don’t Delay. This article included GRADE [21] appraised clinical pathways to achieve an early diagnosis of CP [22]. The title of this article in itself a call for early detection of CP. 

## 3. Current State of Early Diagnosis of CP Globally

Despite the long-standing voices calling for the early diagnosis of CP, diagnosis still occurs relatively late. Currently in Australia only 21% of infants receive a diagnosis by 6 months of age and 52% have a diagnosis made after one year of age [23]. Early diagnosis allows access to CP specific early interventions. We acknowledge that currently there is no cure for CP, and false hope should not be communicated, but we believe it is important for more research to be conducted in order to identify paradigm shifting treatments. In the last decade preventative treatments have been discovered and implemented with substantial population effects. The prevalence of CP has declined from 2.1 (95% confidence interval [CI] 2.0–2.3) in 1995 to 1.4 (95% CI 1.3–1.5) per 1000 live births in Australia [23]. In addition, the motor severity of CP has also decreased over the past decade in Australia [24]. These population trends indicate that changes in preventative neonatal care are successful and that more research is worthwhile. Increasing evidence of the protective effects of neuroprotective and neuroregenerative treatments, including stem cell treatments [25], may deliver improved outcomes in the future. 

Early diagnosis also allows for timely psychological support for parents. It has been reported most parents suspect CP before receiving a diagnosis [26]; and all parents in one qualitative study found early diagnosis beneficial [27]. Empirical evidence verifies that parental interventions (such as Acceptance and Commitment Therapy) improves psychological adjustment in parents of older children [28], and efficacy is now being confirmed in parents of infants under two years. It is hypothesized that early support for parents of infants with CP will be protective because home based parent support after preterm birth conferred lower rates of parental anxiety (odds ratio (OR): 0.28, 95% CI 0.11–0.71) and depression (OR: 0.27, 95% CI 0.08–0.93) [29]. 

It is well established [4,22,30] and continues to be argued [31] that CP is a clinical description rather than an etiologic diagnosis. As such no biomarkers exist for the diagnosis of CP, which is a long-standing barrier to early diagnosis. In 2017 an international clinical guideline which systematically reviewed evidence on tools for the early, accurate diagnosis of CP was published [9]. The guideline clearly states that CP can often be accurately diagnosed under the age of six months and even as early as three months corrected age using a combination of assessment tools with strong predictive validity coupled with clinical reasoning [9]. A diagnosis under six months of age is much earlier than the reported average age of diagnosis of 12–24 months [9] in high income countries. In the guideline, the three tools with the best predictive validity for detecting CP in high risk infants under 5 months of age are: (1) GMA (sensitivity 98% (95% CI 74%–100%); specificity 91% (95% CI 83%–93%) at fidgety age [32], (2) MRI at term equivalent age (sensitivity 86%–100%, specificity 89%–97% [32]), and 3) HINE (sensitivity at 3 months 96%, specificity 85%, CI not reported [33]). The use of CUS in a Danish cohort has shown to be one factor which decreases the age of diagnosis (8.4 months with CUS versus 13.2 months without CUS, *p* < 0.001) [34]. Accurate diagnosis using evidence-based tools is still possible over five months of age, when the GMA cannot be reliably scored, and evidence based diagnostic pathways exist [9]. This pathway is important as half of children with CP do not have newborn detectable risks such as prematurity [22], and often miss the window in which GMA can be completed.

One barrier to an early diagnosis is a concern of making a false positive diagnosis with regards to a normal outcome or other etiology. Population data indicates less than 5% of suspected cases are confirmed as not CP at 5 years of age [23]. A diagnosis of CP can and should be made in conjunction with other diagnoses if a child meets criteria for CP [31,35]. The theme of “outgrowing CP”, proposed initially by Nelson in the 1980s [36], remains topical [37,38]. A lack of perinatal adverse event (odds ratio (OR) 4.1, 95% CI 1.6–10.7) and normal MRI findings (OR 7.8, 95% CI3.8–16.1) have been reported as more likely to occur in infants who do not meet criteria for CP at age 5 years when after a previous diagnosis had been made [37]. However, given tools with stronger predictive psychometrics are now being implemented in practice, it is probable that the small rate of false-positive cases of CP will decrease even further in the future. 

The guideline also sets out evidence-based pathways for early diagnosis in low- and middle-income countries (LMIC) where tools such as neuroimaging are not standard care. The potential for impact on decreasing the age of diagnosis in LMIC contexts is enormous. For example, the average age of diagnosis in Bangladesh is currently five years of age [39]. Late diagnosis has been identified as one reason Bangladeshi children have limited access to early intervention [39]. To aid a change in practice, the feasibility of implementing the international guideline has also been confirmed [40]. We are now at the juncture where evidence-based early diagnosis tools exist that are feasible to implement in a range of contexts, which may make early diagnosis the new norm. 

## 4. Understanding How the Definition of CP Relates to Infants: A Barrier to Early Diagnosis? 

The way in which some of the essential elements of the 2007 consensus definition of CP are conceptualized in infants may have diminished clinicians’ confidence in making an early diagnosis of CP.

### 4.1. Permanent Disorder of the Development of Movement and Posture 

A core feature of CP is impaired motor control, which is seen in altered movement and postures leading to abnormal motor functioning [4]. Understanding what impaired motor control and functioning look like in an infant under the age of six months can be difficult, but some new tools exist. For example the Hand Assessment of Infants (HAI) [41] gives reliable information on detecting if hand function is developing as expected [42] from three months of age in infants with unilateral CP. Standardized, norm referenced assessments of gross and fine motor development, for example the Peabody Developmental Motor Scales [43] and Bayley Scales of Infant and Toddler Development [44], are often not sensitive enough to detect a motor delay until after six months of age in infants with CP, especially in mild cases [9]. Moreover a motor delay can also occur in many other diagnoses, increasing uncertainty. The lack of specificity of available standardized motor assessments makes it difficult for clinicians to be certain that any motor delays observed fit the criteria for CP.

### 4.2. Activity Limitation

An integral concept defining CP is that the motor disorder must cause activity limitation. It was agreed in 2007 that children with a brain lesion but without activity limitations in terms of motor function should not meet the criteria for CP [4]. This concept stands today. The World Health Organization defines activity limitations as “difficulties an individual may have in executing a task or action” [45]. Understanding what constitutes activity limitation for a three or six-month-old can be difficult to ascertain. Younger infants have a relatively limited motor repertoire from which to assess delay or impairment compared to their typically developing peers [43]. In addition, the age range for typical achievement of some motor skills is broad [46]. Typically, activity limitation is associated with a delay in milestone attainment; however, many gross motor milestones are not reached until after six months (e.g., independent sitting). This is well into the critical period for optimizing neuroplasticity. The early spontaneous motor repertoire of infants has been described including: reaching movements of the arms, mutual manipulation of fingers and leg lifting [47]. An adjunct to the basic scoring of general movements called the motor optimality score exists [48]. A reduction in the amount of fetal movements is a cause for concern during pregnancy [49]. It is postulated that very early movement repertoire could be considered the “activity” of a newborn or young infant. Our knowledge of the early motor repertoire and how this might be impaired in infants with CP is still emerging (See Einspieler et al. in this issue). Waiting for an infant to have a delay of a classical motor milestone such as sitting to prove an activity limitation exists may be too late.

### 4.3. Non Progressive Brain Lesion

According to the current definition, CP is attributed to a non-progressive brain lesion, however the condition itself is not unchanging [4]. Progressive musculoskeletal impairments including contracture and bony torsion develop throughout childhood and adolescence [4]. It is universally agreed that progressive brain lesion disorders do not meet criteria for CP; however, modern understanding of neuroplastic potential of the brain, particularly the infant brain, means that brain lesions are no longer considered unchangeable [50,51]. In practice making a diagnosis of CP without neuroimaging findings which complement the clinical presentation is done cautiously until the etiology of the presenting condition is better understood. Normal findings on MRI are reported in 10%–15% of children with CP [15,52,53,54]. In addition progressive disorders with a similar clinical presentation to CP exist such as hereditary spastic paraplegia. Ruling out differential diagnoses does create a barrier to making an early diagnosis, and clinicians are right to investigate the cause of the presenting condition. Furthermore, interpretation of brain MRI is also age dependent. Myelination of the infant brain occurs over the first two years of life and MRI findings need to be interpreted accordingly [55]. Mild lesions on both CUS and MRI may be missed in MRI under the age of two years as myelination is not yet complete. 

The utility of sequential cranial ultrasonography in the preterm to term infant is well established [14,56]. CUS has 74% sensitivity (95% CI 63%–83%) and 92% specificity (95% CI 81%–96%) for predicting CP [32]. In the term infant and beyond, MRI should always be considered especially when the history does not match the clinical findings [32]. This is true for any infant in which there are no risk factors for CP. In those infants with risk factors for CP, MRI can help in not only establishing a diagnosis of CP but in determining why the infant has CP, which is equally important. A brain MRI can, in many cases, help to establish causation and differentiate between those with structural abnormalities, vascular events, and metabolic or genetic syndromes. Suspicion regarding etiology should escalate for those children in whom the MRI is normal and should prompt the physician to broaden testing especially if genetic or metabolic causation is suspected. In these infants, abnormal genetic findings, explaining causal pathway to CP, will lower the risks for false positives.

The 2007 consensus definition of CP [4] stands when applied to infants. However, some concepts within the current definition may need re-shaping in terms of how they relate to infants. For example, what is a disorder of movement and posture, and what is an activity limitation in a three or six-month-old infant? 

## 5. Classification of CP 

CP is a heterogeneous condition in terms of etiology, motor type, and severity of impairments. Consequently, CP is described using different classifications primarily motor type, topography, and motor severity [4]. Motor Type: Motor types of CP include spastic (85%), dyskinetic (7% (which includes dystonia and choreoathetosis)), and ataxic (4%) [23,57]. The predominant motor type hypotonic CP (3%) is recognized in Australia [23], but there is international disagreement about whether hypotonic CP is truly CP [23,57]. Topography: The spastic motor type is classified topographically as (i) unilateral (hemiplegia (40%–60%)) affecting one side of the body or (ii) bilateral affecting both sides of the body. Bilateral spastic CP includes: diplegia (10%–36%), with lower limbs more affected than upper limbs; and quadriplegia (24%–31%) [23], with trunk and all four limbs affected. Dyskinetic, ataxic and hypotonic predominant motor types are not classified topographically. Motor Severity: Prediction of motor severity of CP over the age of two is well established using the Gross Motor Function Classification System (GMFCS), which indicates a child’s level of gross motor function and mobility [58], and CP motor development curves [59]. Both tools are recognized tools used to predict long term mobility [60]. Of all of the classifications only GMFCS is known to be reliable [61,62]. Despite classification of motor type and topography being unreliable [1,22] they have a place in guiding targeted evidence-based therapies [22]. These classifications are also used in CP registers to describe cohorts [23,57]. 

### 5.1. Is Classification One Type of Pattern Recognition? 

Pattern recognition is one strategy used by clinicians during the initiation and refinement of a diagnosis [63]. For example, neuroanatomical localization in neurology is used to help make sense of a clinical presentation. In older children and adults with CP it is postulated that one form of pattern recognition could be recalled and described using different classifications. For example, a clinician would recognize two very different patterns if they met (a) a four-year-old child with spastic hemiplegia, GMFCS level I or (b) a four year old child with dyskinetic CP, GMFCS IV. In older children recognition using motor type, topography, and severity is interconnected with the diagnosis of CP. 

### 5.2. Rethinking Classification Pattern Recognition in Infants With CP—It Will be Different to Older Children 

Overt clinical signs of CP seen in older children often do not begin to emerge until after six months of age [64] and neurological signs seen in infants with CP differ from those seen in older children. For example, spasticity is often not able to be detected under the age of 12 months and may even emerge later. This emergence of neurological signs is thought to be a consequence of ongoing myelination of the developing brain. It is not fully understood at what age the overt signs of different motor disorders as they are known in older children, including spasticity, dyskinesia, ataxia and hypotonia, develop although it is recognized that neurological signs in infants emerge and change over at least the first two years [9]. For example, an infant may present with hypertonia at a young age which does not develop to be spasticity at a later age. 

When the clinical scenario is not clear in an infant, one might wait for a known classification pattern of CP to emerge in order to make a diagnosis. In this way, pattern recognition using classification of CP as it presents in older children can become a barrier to making a diagnosis in infants. However, new patterns can be established and recognized. For example, an infant may present with absent fidgety general movements at fidgety age and a brain lesion indicating motor impairment but with no presenting spasticity. In this scenario, although at this young age there is not a need for spasticity management, recognizing the pattern, making a diagnosis and referring to early motor interventions [65,66] is appropriate before spasticity appears. In addition, pattern recognition is used in the GMA using gestalt perception [16,47], described as a powerful instrument for movement analysis [16]. 

In rethinking classification pattern recognition in infants with CP, emerging motor tests such as Hand Assessment of Infants (HAI) [67] and possibly tools which have the best predictive validity in predicting later CP such as GMA, HINE, MRI, and motor tests [9] are likely to add the most information to the pattern description. In the future a better understanding of which tools most accurately predict early classification of motor type, topography and severity may aid the recognition of different patterns in infants with CP. This in turn will increase confidence in making an early diagnosis of CP.

## 6. Does Diagnosis and Classification of CP Need to Occur Simultaneously in an Infant?

When the first extensive classification system in CP was published by Minear and the American Academy for Cerebral Palsy in 1956 [68], it was recommended classification make up a part of the diagnosis of CP in order to fully understand the presentation and needs of a child [68]. At this point, Minear warned against describing the motor type of CP too early in infancy due to the changing neurological signs and symptoms at an early age [68]. Some clinicians continue to raise concerns about making a firm diagnosis of CP when a child cannot be clearly classified, (again, consider the example of an infant who presents with absent fidgety general movements and congruent findings on MRI, but does not present with spasticity at six or even nine months of age). This lack of clarity around classification can add uncertainty around making a diagnosis. Recommendation 1.0 of the GRADE appraised international clinical guideline states “The clinical diagnosis of CP can and should be made as early as possible” [9]. The guideline has separate recommendations for the early accurate detection of motor severity and motor type and topography. These recommendations clearly allow for a diagnosis of CP to be made at a different time, namely earlier, as there is better predictive validity of tools for predicting CP than tools which predict early classification of motor type, topography and severity.

## 7. Current State of Early Classification of CP under Two Years of Age

Although the process of diagnosis and classification should be uncoupled, the earliest possible accurate classification of motor type and topography is recommended in the international guideline [9]. This is to direct parents to specific early interventions such as baby constraint induced movement therapy [66] and other emerging rehabilitation programs targeted at early motor and cognitive development [65,69]. In addition, prognostic questions from parents are well recognized [70]. The first questions parents often ask, no matter what the age of diagnosis, are: “What will the future for my child look like?” and “What can we do now?” Early prognostic information on motor severity will help to answer parent’s questions about their child’s future mobility. Evidence exists for several tools to help understand early motor type, topography, and severity, although the quality of evidence is low to moderate [9]. 

### 7.1. Early Classification of Motor Type

Determining motor type in infants with CP with accuracy is difficult; however, some clinical signs of motor type exist [9]. The location, nature and extent of a brain lesion as seen on MRI is related to different motor types [9,14,53]. For example, grey matter injury (basal ganglia damage) is common in dyskinetic CP [14,52], while focal vascular insults are predominantly seen in spastic hemiplegia [53]. GMA trajectories, analysis of movement repertoire and neurological examination can provide information on possible motor type [64]. Trajectories of cramped synchronized general movements over several weeks from term age followed by absent fidgety movements at 3 months is highly predictive of bilateral spastic CP (sensitivity 98%, specificity not reported) [64]. Circular arm movements are often seen in infants later diagnosed with dyskinetic CP [71]. There is very little information published regarding early signs of ataxic CP [9]. This is likely to be a combination of small numbers of infants with the ataxic sub-type (4% [23,57]) and a relatively high proportion (24%–57%) of infants with ataxic CP having a normal MRI [53]. Some tools which exist for the discrimination and measurement of motor type of CP in older children such as the Hypertonicity Assessment Tool [72] are not appropriate for use in a preverbal infant population. A recent systematic review ascertained that there is currently insufficient evidence to show that observable postures or asymmetries can be used as clinical markers for motor type in CP [73], therefore further data on suggested clinical markers is required.

### 7.2. Early Classification of Topography

Early asymmetrical hand use in infants can be an early sign of concern in unilateral CP [9] and asymmetry of hand and digit segmental movements at three months has been suggested as a clinical marker to predict hemiplegia [74]. A model to predict early unilateral CP using the HAI each hand sum score and MRI is being developed with very good results (area under curve, AUC = 0.980) [67]. The model uses a number of clinical results and risk factors, although it does not use an outcome of GMA. This model shows the best predictive value for unilateral CP to date, but is not yet available clinically. A HINE score <63 in combination with >5 congruent asymmetries has low quality but emerging evidence for predicting unilateral CP from typical development (sensitivity 91.8% (95% CI 83%–96%), specificity 100% (95% CI 0.95–1.0), median age 15 months) [75]. Term equivalent age MRI findings also have value in predicting topography of CP in both preterm and term born infants [14]. Brain lesion location and extent adds information about unilateral versus bilateral CP [53] with focal vascular insults most common in unilateral spastic CP (24%) and bilateral white matter injury the most common injury seen in bilateral spastic CP (31%–60%) [53]. 

### 7.3. Early Classification of Motor Severity

GMFCS under the age of two years is less reliable than in children older than two years (κ = 0.55 children < 2 years, κ = 0.75 > 2 years) [58]. Infants should be reclassified at age two, as infants often change GMFCS level [76]. The original GMFCS was intended for infants aged 18 months to six years, with the youngest age band “before 2nd birthday” [58], therefore it should probably only be used between the ages of 18 months and two years. There is limited data for gross motor development curves for children under the age of two, and no data under the age of one [59,77]. 

The use of HINE cut off scores and MRI are recommended for use with infants to predict motor severity [9], however the strength of these recommendations is only conditional based on low quality evidence. Most HINE cut off scores are available for infant cohorts with newborn detectable risks e.g., prematurity and encephalopathy [78]. HINE cut off scores can help predict if an infant is likely to be ambulant. For example, a cut off score of 50 at three months and 52 at six months corrected age predicts independent walking at two years in preterm infants (sensitivity 93%, specificity 100%, CI not reported) [79]. A HINE score < 40 at any age under 14 months is emerging as predictive of likely GMFCS III-V (non-ambulant CP) [9,78,80,81]. A HINE score of 40–60 indicates likely GMFCS I-II (ambulant CP) [9,82]. It is recognized that HINE cut off scores need to be used in the clinical context, for example in combination with MRI findings, not as a standalone tool when predicting severity. 

MRI findings predictive of non-ambulant CP include grade IV bilateral parenchymal hemorrhage, grade III bilateral cystic periventricular leukomalacia (PVL), brain maldevelopment, and basal ganglia injury [9,14,54]. Ambulant CP (GMFCS I-III) is more likely after non-cystic PVL (sensitivity 78%, specificity 96%, CIs not reported [83]) and moderate to severe white matter injury [9,53]. However, a proportion of children with deep grey matter injury leading to dyskinetic CP may also fall into the “mild to moderate” or ambulant group. There is also a higher rate of normal findings (17%) and focal vascular insults (14%) on MRI in children with mild CP (GMGFCS I-II) [53]. Visual function influences if a child with CP will be able to walk independently, with children who are not blind 2.4 times more likely to walk independently than those who are blind [84], although having seizures did not change the odds of being able to walk independently [84]. 

Lastly specific motor milestones such as sitting independently (OR: 12.5, 95% CI 5.8–27.2), pull to stand (OR: 18.5, 95% CI 13.4–60.4) [84], pushing up in prone, and rolling supine to prone [85] (sensitivity and specificity not given), as well as the rate of motor skill acquisition, have been shown to be predictors of ambulation of infants with CP at ages three to five years. It has long been known [10] and corroborated in several studies [84,86] that sitting independently by 24 months is a predictor of later ambulation with or without support. However, earlier accurate predictors of future ambulation are required when a diagnosis has been made in the first year of life. Despite the conditional strength of evidence, it is recommended that early prognostic information regarding severity be given to parents with caution [9].

## 8. How Can Early Detection of CP be Improved?

Implementation strategies lower the average age of diagnosis requires a whole of health system approach. For example, ensuring that all neonates admitted to a NICU who have abnormal neuroimaging predictive of a motor impairment or other known risk factors for CP receive routine GMA and HINE completed in follow up programs. Translational research programs which aim to implement such strategies have been developed [87]. Infants with CP who do not have newborn detectable risks, and are seemingly healthy at birth, are less likely to be followed up. Specific strategies for identifying these infants and administering best practice tools is needed. Use of a smartphone app by parents to record GMA video has been shown to be feasible and acceptable to parents in high risk patients [88]. Automated GMA scoring software programs are under development. While currently using automation to detect general movements is not as accurate as human gestalt perception [89] further work in this area is warranted. Automated technologies may make universal screening for CP using GMA feasible. Newborn hearing screening [90] can be used as a precedence for universal screening. Although, it is recognized that considerable human resources would be required to relay assessment results compassionately if universal screening using GMA was implemented.

## 9. Conclusions

There have been calls in the literature for the earliest possible diagnosis of CP to access early diagnosis specific intervention since William Little first described CP in 1867, however currently only 21% of infants have a diagnosis under six months in Australia. An international clinical practice guideline giving clear pathways to evidence-based tools for accurate early diagnosis has been published. With coordinated international efforts aimed at decreasing the age of diagnosis, we are now in an era where this situation is likely to change. Identifying barriers to clinicians feeling confident to make an early diagnosis of CP will help to ensure we do not disadvantage children by a late diagnosis denying them CP specific early intervention opportunities aimed at optimizing future outcomes. Understanding which tools have the best evidence for the early classification of CP will best guide CP specific early interventions and help to give parents early and accurate predictive information about their child’s future.
